# Gut Microbiota Dynamics in Relation to Long-COVID-19 Syndrome: Role of Probiotics to Combat Psychiatric Complications

**DOI:** 10.3390/metabo12100912

**Published:** 2022-09-27

**Authors:** Maha F. Alenazy, Haya I. Aljohar, Ashwag R. Alruwaili, Maha H. Daghestani, Mona A. Alonazi, Ranyah S. Labban, Afaf K. El-Ansary, Hanan A. Balto

**Affiliations:** 1 Department of Physiology, College of Medicine, King Saud University, Riyadh 11682, Saudi Arabia; 2 Department of Pharmaceutical Chemistry, College of Pharmacy, King Saud University, Riyadh 11495, Saudi Arabia; 3 Department of Radiological Sciences, College of Applied Medical Science, King Saud University, Riyadh 11595, Saudi Arabia; 4 Department of Zoology, College of Science, King Saud University, Riyadh 11495, Saudi Arabia; 5 Department of Biochemistry, College of Science, King Saud University, Riyadh 11495, Saudi Arabia; 6 General Administration of Nutrition, Ministry of Health, Riyadh 11595, Saudi Arabia; 7 Senior Scientist, Central Research Laboratory, Female Campus, King Saud University, Riyadh 11595, Saudi Arabia; 8 Department of Restorative Dental Sciences, College of Dentistry, King Saud University, P.O. Box 62645, Riyadh 11595, Saudi Arabia

**Keywords:** long COVID-19, gut microbiota, gut–organ axis, psychiatric disorders, probiotics

## Abstract

Increasing numbers of patients who recover from COVID-19 report lasting symptoms, such as fatigue, muscle weakness, dementia, and insomnia, known collectively as post-acute COVID syndrome or long COVID. These lasting symptoms have been examined in different studies and found to influence multiple organs, sometimes resulting in life-threating conditions. In this review, these symptoms are discussed in connection to the COVID-19 and long-COVID-19 immune changes, highlighting oral and psychiatric health, as this work focuses on the gut microbiota’s link to long-COVID-19 manifestations in the liver, heart, kidney, brain, and spleen. A model of this is presented to show the biological and clinical implications of gut microbiota in SARS-CoV-2 infection and how they could possibly affect the therapeutic aspects of the disease. Probiotics can support the body’s systems in fighting viral infections. This review focuses on current knowledge about the use of probiotics as adjuvant therapies for COVID-19 patients that might help to prevent long-COVID-19 complications.

## 1. Introduction

Severe acute respiratory syndrome coronavirus 2 (SARS-CoV-2), the pathogen causing coronavirus disease 2019 (COVID-19), has produced morbidity and mortality at an extraordinary rate worldwide [1]. Scientific and clinical proof is evolving on the sub-acute and lasting effects of COVID-19, which can disturb various organ systems [2]. Primary reports propose long-term effects of SARS-CoV-2 infection, such as fatigue, cognitive disturbances, chest pain, dyspnea, arthralgia, and decline in quality of life [3,4]. In 2021, the finding of an online survey conducted from 6 September 2020 to 25 November 2020 of 3762 participants with confirmed, and 2742 participants with suspected, infection suggests that the morbidities of COVID-19 have been greatly overlooked. They reported that after seven months, many patients had not yet recovered, still suffered from multisystem disabilities, and had not returned to their daily functioning and baseline health [5]. Cellular injury, a tough innate immune response with remarkably high pro-inflammatory cytokine production, and a pro-coagulant state triggered by SARS-CoV-2 infection may contribute to these complications [6,7].

Liu et al. [8] classified the complications of long COVID-19 into respiratory (runny nose, nasal congestion cough, sputum, and shortness of breath), neurological (loss of taste, loss of smell, dizziness, anxiety, headache, cognitive problems, sleep disturbance, poor memory, depression, and blurred eye), gastrointestinal (diarrhoea, nausea, abdominal, and epigastric pain), dermatological (hair loss), musculoskeletal (muscle and joint pain), and fatigue [8].

Cumulative evidence has revealed that gut dysbiosis is associated with the severity of COVID-19 infection and long-lasting multisystem complications after disease recovery [9]. Several studies have made significant contributions to understanding the gastrointestinal (GI) tract in the context of long-COVID-19 syndrome. This is related to the much higher expression of SARS-CoV receptors (ACE2) in the GI tract and the impairment of gut microbiota in subjects infected with SARS-CoV-2 [10,11]. Patients with COVID-19 showed significant alterations in fecal microbiomes compared with non-COVID-19 controls, characterized by a higher abundance of opportunistic pathogens and lower growth rates of healthy or good bacterial strains [11,12]. Several gut commensals with known immunomodulatory effects, such as *Faecalibacterium prausnitzii*, *Eubacterium rectale,* and *bifidobacteria*, were found to be lower in the fecal samples of COVID-19 patients and remained low up to 1 month after disease recovery [13].

Zuo et al. [12] reported that the SARS-CoV-2 virus load in fecal samples of patients is inversely correlated with Bacteroides dorei, *B. thetaiotaomicron*, *B. massiliensis*, and *B. ovatus* [12]. In this context, it is exciting that Bacteroides species can downregulate intestinal ACE2 expression when monocolonized in the intestines of germ-free mice [14]. In particular, *B. thetaiotaomicron*, a dominant anaerobe commensal bacterium in the human gut, is well-known to have anti-inflammatory properties and ameliorate mucosal barrier function in rats with chemically induced colitis [15,16]. Collectively, a reduction in *B. thetaiotaomicron* could induce ACE2 expression and reduce anti-inflammatory activity, which may enhance SARS-CoV-2 infectivity and intestinal/systemic inflammatory tone.

## 2. Oral Health as Contributor to Long COVID-19

It was observed that the severity of COVID-19 symptoms significantly increased in patients with poor oral health and decreased in those with good oral health status. Moreover, the correlation of oral health with recovery period and C-reactive protein (CRP) values is significantly inverse, showing that poor oral health is correlated with increased values of CRP and delayed recovery period [17]. Promoting optimal oral health and raising oral (self) care awareness among the public via oral health professionals is, thus, encouraged [18,19].

After recovering from the acute phase of COVID-19 and being discharged from hospital care, it is more important to emphasize how to achieve oral health for people in an outpatient setting in a way that is both applicable and will not result in care-related complications [20]. Gherlone et al. (2021) [21] noted the development of changed taste and smell, salivary gland ectasia, white tongue, dry mouth, facial muscle weakness and dysesthesia, oral ulcers, temporomandibular disorder, and other new abnormalities in a population of people who had had COVID-19 [21]. According to many observations, the symptoms of long COVID-19 in the oral cavity are associated with a decrease in the body’s immunity, higher stress, and the lower general health of the patient [22,23]. 

Patients with COVID-19 often undergo intubation, aided exterior ventilation, and tracheostomy [24]. These processes result in hyposalivation, which worsens various pre-existing grievances in the oral cavity and can induce bacterial secondary pneumonia [25]. SARS-CoV-2 appears to cause tropism for nerves and damage to sensory neurons has been postulated in the repeated occurrence of anosmia [26]. Neuronal damage may also disturb facial muscle tone and weaken the secretory function of salivary glands. Gherlone et al. (2021) [21] reported that the examination of the oral cavity of COVID-19 patients after effective viral clearance proved the direct cytopathic action of the virus on infected cells of the oral tissues. It is more probable that oral associations arise as a consequence of the patient’s inflammatory response, which is responsible for most morbidity and mortality in COVID-19 [21]. In agreement with a role of the early innate immune response, Zangrillo et al. (2020) [24] found a highly significant relationship between salivary gland ectasia, the levels of CRP, a marker of systemic inflammation, and lactate dehydrogenase (LDH), a biomarker of general necrosis, at the beginning of clinical symptoms. LDH also acted as an independent predictor of salivary gland ectasia in multivariate analysis. LDH and CRP have been suggested as measures of COVID-19 severity [24]. Therefore, it is interesting to highlight the association of salivary gland abnormalities with long-COVID-19 complications. Interestingly, anti-SARS-CoV-2 antibodies were found to be still detectable in COVID-19 patients’ saliva for at least three months after symptom onset [27,28], indicating the involvement of the oral cavity as an immune site during COVID-19. Therefore, it is recommended to perform an extensive intraoral examination in recovered COVID-19 patients to find any related oral manifestations. 

## 3. Role of Gut Microbiota–Organ Axis in Long-COVID-19 Multi-Organ Dysfunction

### 3.1. Gut–Liver Axis and Long COVID-19

**Figure 1:**
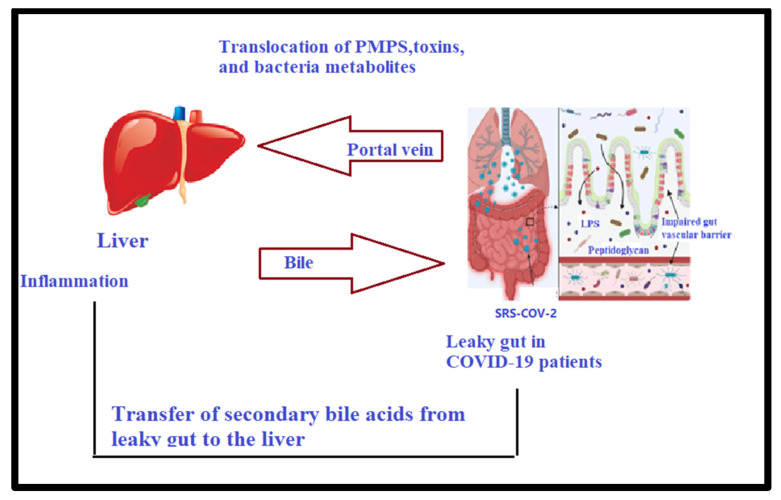
Gut–microbiota–liver axis in COVID-19 patients: role of dysbiosis and leaky gut in long-lasting liver complication through PAMPs, secondary bile acids, toxins, and pathogenic bacteria metabolites.

The gut–liver axis refers to the bidirectional pathway between the gut and its microflora and the liver, which is based on the integration of signals produced by genetic, dietary, and environmental influences. This mutual interaction is established by the portal vein, through which gut-derived products are directly transported to the liver and the liver feedback rout of bile and antibody discharge to the gut (Figure 1).

Two recent studies suggested that metabolic-dysfunction-associated fatty liver disease (MAFLD) is a major risk factor for progression to severe and long-lasting COVID-19. Both studies prove that patients with signs of MAFLD had a higher risk of respiratory disease progression than patients without MAFLD, with a much higher risk in younger than older COVID-19 patients [29,30]. Assante et al. (2021) [31] suggested that the increased risk observed in patients with MAFLD could be related to the effect of SARS CoV-2 infection on the gut, which worsens intestinal permeability and mucosal inflammation, thus, exacerbating systemic immune dysfunction as a feature of severe COVID-19 [31]. Certainly, this process may also clarify the higher risk for COVID-19 progression in obesity, T2D, and even IBD, which are associated with altered gut microbiota, mucosal inflammation, and increased intestinal permeability.

Numerous studies have stated that GI symptoms, such as diarrhea, vomiting, and abdominal pain, are common in patients with COVID-19 and that the severity of GI symptoms increases in coincidence with respiratory problems and liver dysfunction [32,33]. Additionally, ACE-2 SARS CoV-2 receptors have been found to be expressed on enterocyte cells, as the high levels of SARS CoV-2 viruses in feces suggest that the gut is a plausible site of viral infection and inflammation. The trans-membrane serine protease 2 that is used for SARS-CoV-2 entry is also widely expressed in gut cells [34,35]. Based on this, the increased expression of viral entry receptors in the GI tract, as well as the early onset of GI symptoms, implies that GI abnormalities may result from the direct viral worsening of leaky gut, rather than being a consequence of a secondary immune–pathogenic response to the upper respiratory tract infection.

The clinical presentation of GI symptoms that are positively correlated with biomarkers of liver dysfunction supports the concept of the increased transmission of pathogen-associated molecular patterns (PAMPs) to the liver [32].

SARS-CoV-2 infection disrupts the gut barrier and leads to the elevation of systemic bacterial lipopolysaccharide and peptidoglycan and it serves to enhance systemic inflammation. Therefore, leaky gut and microbial dysbiosis could contribute to the development of cytokine storm in patients severely ill with COVID-19.

Based on this, treatments that have been developed to treat leaky gut, such as probiotics and prebiotics for gut mucosal protection/regeneration, could minimize the number of patients with MAFLD/obesity/T2D that progresses to severe and long-lasting COVID-19. Moreover, drugs that disturb intestinal microbiota, such as antibiotics, should be avoided during SARS CoV-2 viral infection.

### 3.2. Gut–Heart Axis in Long COVID-19

It is well documented that gut microbiota alterations and reductions in gut bacterial diversity are common in patients with heart failure and coronary artery disease [36].

A dysfunctional gut barrier could ease the passive leakage of bacterial products, among which is the passage of pro-inflammatory lipopolysaccharides (LPSs) to the blood, which could contribute to systemic inflammation through inflammasome activation. This has been proven through the remarkable increase in plasma levels of LPS binding protein (LBP) as a predictive biomarker of high cardiovascular risk in old-aged men [37]. Interestingly, gut leakage and inflammasome activation have been found to be positively correlated with troponin as a marker of myocardial damage.

In an attempt to understand the role of the gut microbiota–heart axis in long-COVID-19 syndrome, it is interesting to mention that a considerable percentage of hospitalized COVID-19 patients have cardiac problems [38]. Earlier cardiovascular disease (CVD) and risk factors for CVD, such as obesity, appear to be key risk factors for developing severe and long-lasting COVID-19 complications [38,39]. Yet, a high proportion of COVID-19 patients have cardiac involvement without previous CVD [40]. 

**Figure 2:**
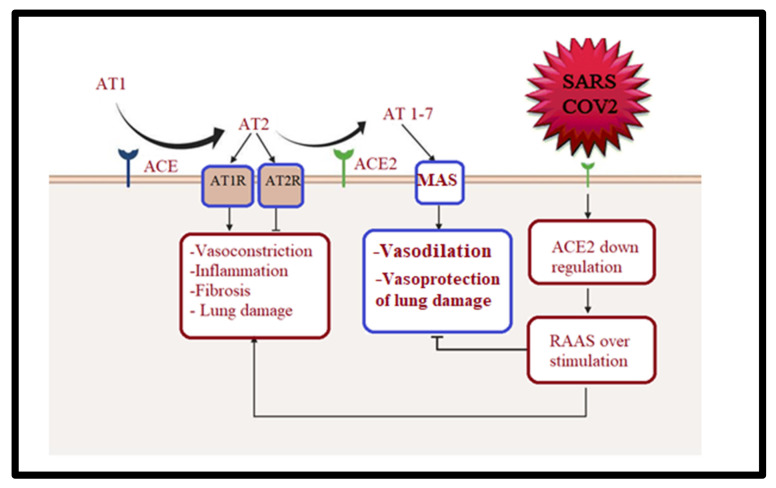
Potential impact of the gut–heart axis in long-COVID-19 patients.

Cardiac issues have also appeared as a substantial and life-threatening problem in COVID-19 patients, ranging from myocardial infarction (MI) and myocarditis to pulmonary hypertension with cardiac stress [41,42]. The mechanisms underlying this cardiac involvement are not clear. Angiotensin converting enzyme (ACE2) is expressed in several organs and, in addition to the lung, heart, and kidney tissues, ACE2 is also expressed in the gut, where ACE2 expression areas in enterocytes may serve as sites for SARS-CoV-2 entry and prompt gut infection [43]. The downregulation of the anti-inflammatory and cardio-protective angiotensin (AT)-1-7 pathway, secondary to the downregulation of ACE2, the SARSCoV-2 receptor, directs the infection of the myocardium through ACE2-expressing cardiac cells, leading to cardiac inflammation [44] (Figure 2).

Hoel et al. (2021) [45] suggested that long-term follow-up with cardiac imaging, in combination with microbiota analyses from the gut compartment, represent the necessary next steps to further test the potential impact of the gut–heart axis in long-COVID-19 patients [45]. 

### 3.3. Gut–Kidney Axis in Long COVID-19

**Figure 3:**
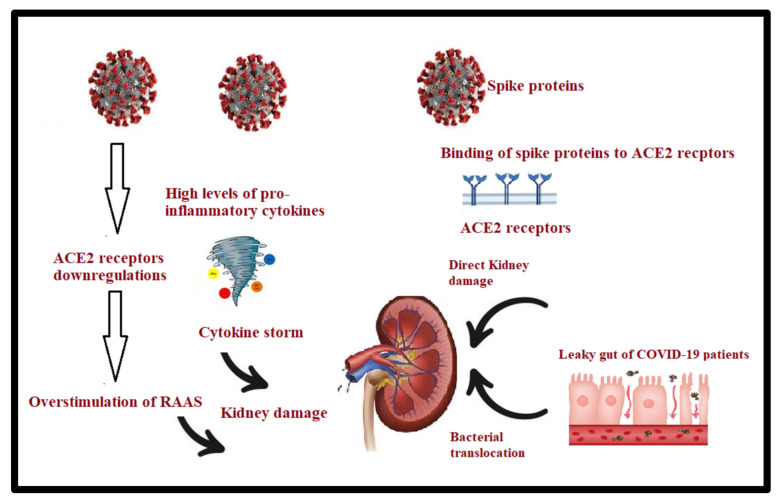
Role of leaky gut and dysbiosis in chronic kidney damage as a long-COVID-19 complication.

The pathogenic interconnection between the gut microbiota and kidney diseases is referred to as the gut–kidney axis and it appears to be involved in a wide range of clinical manifestations, such as chronic kidney disease (CKD), acute kidney injury (AKI), and hypertension. In the case of leaky gut, the passage of viable bacteria frequently occurs from the gut to other extra-intestinal locations, such as the kidneys. This bacterial translocation may be concomitant with dysbiosis, the overgrowth of pathogenic bacteria, and a low host immune system [46,47]. The gut microbiota produces many toxins and uremic solutes, such as p-cresyl sulfate (PCS), indoxyl sulfate, and trimethylamine (TMA) N-oxide, in the case of chronic kidney disease (CKD). On the other hand, increasing urea levels could lead to alterations in the gut microbiota [48] (Figure 3). Uremic toxins may cause fatigue, mineral bone disorders, neurological disorders, and cardiovascular impairment in CKD patients [48].

In an attempt to understand the role of gut dysbiosis in long-lasting kidney problems associated with COVID-19, acute kidney injury (AKI) is commonly addressed as a complication among patients with COVID-19. In addition to pre-existing CKD being associated with severe illness or death in COVID-19 [49], it is noteworthy to address the different pathways through which SARS CoV-2 can access the kidney through the AEC2 receptors and induce clinical manifestations. It is broadly accepted that the virus can directly enter the kidneys and replicate, leading to dysfunction [50], and that it affects the kidneys through local disturbance in the renin–angiotensin–aldosterone system’s (RAAS) homeostasis [51]. Kunutsor and Laukkanen (2020) [52] reported that groups with higher prevalence of pre-existing CKD might be prone to higher incidences of AKI [52]. Emerging evidence also suggests that renal manifestations of COVID-19 are associated with increased risks of long-lasting severe COVID-19-related kidney complications [53]. 

In dysbiotic COVID-19 patients, the beneficial microbiota (primarily Bifidobacterium and Lactobacillus) disappear gradually and a drop in SCFAs and bile acid levels is observed due to the microbiota alteration and pathogen domination [9,54]. SCFAs and, specifically, butyrate are important energy sources for colonocytes [55] and also play an important role in epithelial integrity. Moreover, the activation of the SCFA receptor GPR109A is linked to the suppression of several proinflammatory mediators [56]. This might explain the remarkable long-lasting complications seen in COVID-19 patients. The authors of [57] reported a decrease in the anaerobic microflora in patients with CKD, while an increase in the aerobic microflora background [58] can be observed, with a predominance of Enterobacteriaceae [59]. All these mechanisms could explain the long-lasting kidney complications in some COVID-19 patients.

Monitoring kidney function during hospitalization for COVID-19 could help in the identification of patients at high risk for worse consequences, aiding in early and more effective intervention.

### 3.4. Gut–Brain Axis in Long COVID-19

Although the major clinical presentations of COVID-19 are related to the respiratory system, they can also distress the brain, initiating acute cerebrovascular and intracranial infections. About 35% of patients and up to 85% of those who become severely ill report neurological symptoms, including headache, dizziness, myalgia, or loss of taste and smell [60].

**Figure 4:**
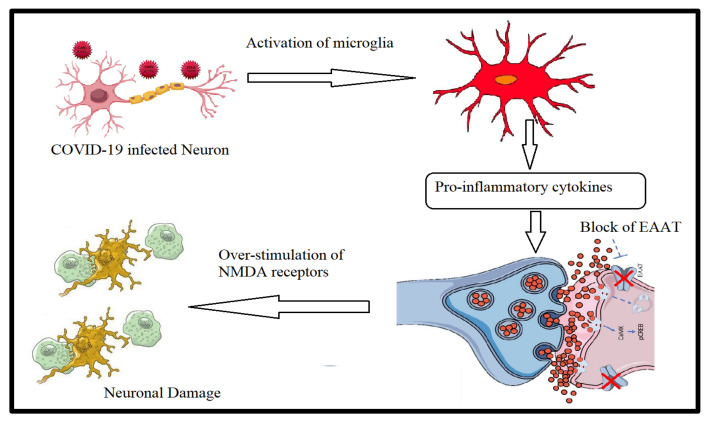
Role of COVID-19 infection in glutamate excitotoxicity as an etiological mechanism of psychological complications.

There are numerous mechanisms through which COVID-19 infection may lead to neurological disorders, as well as structural and functional alterations in the brain (Figure 4). Cognitive troubles are amongst the most commonly reported symptoms, affecting between 10 and 25% of COVID-19 patients and presenting as chronic illness post-SARS-CoV infection [61]. The authors found a consistent pattern of memory deficits in those that had experienced COVID-19 infection, with deficits increasing with the severity of self-reported ongoing symptoms. Moreover, they reported that fatigue/mixed symptoms during the initial illness and ongoing neurological symptoms were predictive of cognitive performance.

There is accumulating evidence that COVID-19 is associated with neural damage, mostly in the presence of neurological symptoms [55,62]. Post-mortem studies of patients who have died from COVID-19 show indications of ischemic injury and evidence of neuro-inflammation as the etiological mechanism [63]. Numerous studies have recorded functional as well as structural deformities, such as hemorrhagic injuries and epileptiform discharges, in different brain areas [64,65].

In relation to neuro-inflammation, the role of glutamate excitotoxicity should be considered as a contributor to long-lasting COVID-19-assocated neurological symptoms. Ahmed et al. (2020) [66] reported that SARS-CoV infection induces a significant increase in the production of pro-inflammatory cytokines and neuronal degeneration as an outcome of glutamate excitotoxicity [66]. Simply, glutamate, as a primary excitatory neurotransmitter in the nervous system, is mainly produced by neurons and discharged in the synaptic cleft, after which it binds to the ligand-dependent AMPA receptor (alpha-amino-3-hydroxy-5-methyl-4-isoxazolepropionoc acid receptor). This helps the access of sodium ions and the passage of the nerve impulse through the post-synaptic neuron, leading to the activation of the N-methyl-D-aspartate receptor (NMDA), which induces the entrance of calcium ions. During the infection of neurons caused by coronavirus, microglial cells produce pro-inflammatory cytokines (TNF-α, IL-1β, and IL-6), which downregulate the glutamate transporter 1 (GLT-1) on astrocytes and pre-synaptic neurons. This will decrease the rate of the efficient re-uptake of glutamate and cause an imbalance in glutamate/GABA neurotransmitters and the overstimulation of NMDA receptors. These events disturb the control of glutamate homeostasis and the overproduction of glutamate in the synaptic cleft induces neuronal excitotoxicity with a significant entrance of calcium, which eventually leads to nerve cell degeneration and loss (Figure 4). 

#### Neuropsychiatric Complications and Gut–Brain Axis

Recent studies have reported that more than one-third of positive patients develop neurological and neuropsychiatric symptoms in the early stages of infection, but other reported cases show such symptoms even after the resolution of COVID-19 [67]. White matter and brain abnormalities, such as hyperintensities and hypodensities, hemorrhages, and infarcts, were found in 34% of the COVID-19 population [55,68]. Severe infections can contribute to delayed neurological central nervous system (CNS) complications. Recent studies have suggested the possible neuroinvasive mechanisms of COVID-19 leading to a neurotropically induced “cytokine storm”, which releases a large number of inflammatory markers [69] and could reactivate immune-mediated processes [70]. Neuroinflammation and any axonal damage are due to viral replication, which causes delayed self-reactive T-cell suppression [71,72]. Different mechanisms suggest that the first approach is a direct viral injury to the CNS via blood circulation or the cribriform plate [73], leading to encephalitis [73,74]. The second stage of CNS damage results from peripheral demyelination [75], which is a host immune response that follows acute infection. Indirect injury is the third mechanism, resulting from systemic circulatory dissemination [76]. The last suggested mechanism is cytokine release as a result of immune response overactivation [77]. These mechanisms could induce delayed nervous system damage and neurological complications, known as long COVID-19 [78]. More reports showing delayed neuro-demyelination following COVID-19 infection [79], confirmed by animal studies, support the theory that SARS-CoV-2 infection crosses the blood–brain barrier and causes acute or delayed CNS demyelination and/or axonal damage [80]. Brain structural abnormalities in postmortem COVID-19 suggest hemorrhagic and posterior reversible encephalopathy syndrome (PRES) [81]. Acute necrotizing encephalopathy (ANE) is a rare COVID-19 complication that has been related to intracranial cytokine storms [82].

Systematic follow-ups in published studies have identified that post-COVID-19 patients after hospitalization suffer from physical and psychological symptom burdens [83]. Psychiatric symptoms are common after a coronavirus infection; many reports have found SARS-CoV-2 infection to be associated with a high prevalence of anxiety [84,85] and patients with these symptoms tend to have more somatic and pain complaints [86]. More recently, attention has focused on many other mental disorders, such as fatigue [67,83], panic disorders, and pain and depressive disorders [87]. Fatigue prevalence in post-COVID-19 patients, at 3–5 months, was reported in 64.2% of cases [88]. Central factors influencing post-COVID-19 fatigue include the levels of dopamine and serotonin [89], which act as neurotransmitters, and many other factors, such as severe changes in axonal conduction velocity or neuronal excitability [90]. Longitudinal studies have found that dysgeusia and hyposmia symptoms are associated with the development of cognitive impairment (CI) after COVID-19 [91,92], with the main involvement of the executive functions, such as flexible thinking, planning, and information processing.

A considerable amount of literature has been published on the neuroimaging findings of multiple brain regions involved in both acute and long COVID-19. 18F-FDG-PET imaging following COVID-19 revealed hypometabolism in the insula, para-hippocampal, fusiform gyri [93], olfactory gyrus, and connected limbic/paralimbic regions [94], as well as frontoparietal hypometabolism [95]. Cortical hypometabolism could be a consequence of white matter (WM) or brainstem damage [95]. Frontoparietal hypometabolism has also been associated with multiple neurodegenerative disorders, which may cause a decline in cognitive function in long COVID-19 [96]. 

Structural MRI studies investigated corticospinal tract and corpus callosum hyperintensities following COVID-19 and found that these are more suggestive of Neuromyelitis-Optica spectrum disorder (NMOSD) [97,98]. The most prominent observation was deep WM abnormalities [99] with diffuse subcortical changes [79,100]. A grey matter (GM) loss in brain regions directly linked to the primary olfactory and gustatory systems can explain the cerebral spread of SARS-CoV-2 [92,101]. Another study yielded opposing results, with higher GM volumes in olfactory cortices, hippocampi, insulas, some regions of the secondary somatosensory and primary auditory cortices, and cingulate gyrus, as well as a general decline in white matter seen via diffusion tensor imaging (DTI), but an increase via fractional anisotropy (FA). The case studies have revealed many other neurological manifestations, such as acute necrotizing encephalomyelitis (ANE) following COVID-19, as a case of an acute CNS injury involving hemorrhage and cavitation [82]. The MRI characteristics of ANE include hyperintensities, with internal hemorrhage on T2-weighted fluid-attenuated inversion recovery (FLAIR) and a ring of contrast enhancement on enhanced images [82].

However, there have also been reports of neuroplasticity, where acute neurological symptoms and CI were resolved at some point from a matter of days to after 10 months [102,103]. There is an urgent need to follow-up COVID-19 survival in order to understand the potential and real long-term consequences, especially for extrapulmonary sequelae.

### 3.5. Gut–Spleen Axis in Long COVID-19

Recently, interest has emerged in the role of spleen function during COVID-19 infection. At the beginning of the epidemic, asplenia as the anatomic absence of the spleen, or spleen dysfunction secondary to disease states, was found to confer a mortality risk comparable to other known risk factors. SARS-CoV-2 was shown to induce a particular tropism for the spleen, possibly through the ACE-2 receptor. Spleen dysfunction was thought to contribute, along with other mechanisms, to B and T cell lymphopenia, which is a typical feature in COVID-19 post-infection [104,105]. Based on the higher abundance of lipopolysaccharides (LPSs) as a product of the Gram-negative bacteria in splenectomized or spleen-dysfunctional patients compared with healthy controls, an altered gut microbiota composition as a major cause of elevated plasma LPS could be related to long-COVID-19 complications [106].

## 4. Immune Perspective on Long COVID-19 and Proposed Mechanisms

There have been increasing numbers of case reports and cited observations documenting chronic symptoms lingering past the actual acute stage of COVID-19 infection [3]. These symptoms are multi-systematic and can be respiratory, cardiovascular, neurological, or hormonal [3]. There are several definitions for long COVID-19 that take into consideration the time it takes to manifest (Table 1). Nonetheless, the general consensus is that long COVID-19 is confirmed when symptoms persist about three months after the clearance of the acute COVID-19 infection [107]. These chronic symptoms of long COVID-19 have been found to include: dyspnea as a common complaint, alongside fatigue, chest pain, palpitations, tachycardia, orthostatic intolerance, headache, depression, insomnia, cognitive impairment, memory loss, changes in taste and smell, joint pain, myalgia, and gastrointestinal symptoms [104,105]. Several studies have investigated the causes of such chronic symptoms of COVID-19 in order to assess the pathophysiology. Mechanisms have been proposed for long COVID-19, but this is still a growing field of interest. More work is needed to clarify its seemingly complicated pathophysiology, as different systems are usually involved based on the clinical symptoms presented.

Examining long-COVID-19′s pathophysiology, immune dysregulation is implicated as the trigger causing other systems to be involved, as the innate immune response is overactivated. This not only disturbs other immune parameters, but also other systems and organs [106,108]. For instance, an overactivated immune response dysregulates the RAAS (renin angiotensin aldosterone system), which has a direct influence on overall body homeostasis [108,109]. This overactivated innate immune response is limited to thrombi formation primarily in micro vessels [108,109]. 

That said, despite other studies that focused on other causes of long COVID-19, considering the neurological and gut microbiota links, this can only be comprehensively understood once it is considered in the immune context, since it is an immune-based dysregulation condition. Examples of such studies that have analyzed other factors include those that have investigated the neurological symptoms of long COVID-19, in order to understand their cause [105]. One suggested cause is the slow regeneration of neurons as the damage to the brain stem can be long-lasting, leading to the neurological symptoms observed in long COVID-19 [105]. 

Nonetheless, many studies have investigated the immunological pathophysiology of long COVID-19, given that it is a viral infection compromising the immune system [110]. For instance, CD8 T cells, which are cytotoxic in nature and mediate adaptive immunity, have been found infiltrating the lungs of long-COVID-19 patients, which supports the diagnosis of T cell dysfunction [110,111]. A mechanism called bystander activation has been proposed for cases in which antigen-presenting cells present antigens to auto-reactive T cells [110,111]. Another example of this auto-reactive T cell dysfunction is the presentation of autoimmune thyroid dysfunction in 15–20% of patients with COVID-19 [112,113]. Another immune cause linked to long COVID-19 is B cell activity dysfunction, as 52% of samples had more severe clinical outcomes, with anti-phospholipid autoantibodies presenting signaling neutrophil hyperactivity [114]. Autoantibodies were also found against interferon, cyclic citrullinated peptides, neutrophils, connective tissues, and the cell nucleus in 10–50% of COVID-19 patients exhibiting long-term symptoms [115]. These findings implicate B cell involvement in long COVID-19’s pathophysiology. This increase in autoantibodies in chronic COVID-19 has been linked to an increased incidence of autoimmune diseases post-COVID-19 infection, such as Sjogren syndromes, lupus erythematosus, and rheumatoid arthritis [116]. More studies have investigated this link and one proposed mechanism is the lymphocytopenia present in severe COVID-19. Nevertheless, many reports have not found such a link between COVID-19 severity and long-COVID-19 symptoms, which further complicates the understanding of the immune pathophysiology of the condition [3,107]. 

More studies have investigated the association between long COVID-19 and immune cellular activity [117,118]. Some reports may appear contradictive but taking into consideration the different definitions for long COVID-19, which entail different timelines, can explain the variation and help us to understand the immune pathophysiology. For instance, some studies report that long COVID-19 is linked to the unresolved hyperinflammation exerted by the renewed B and T lymphocytes during infection [117]. This hyperinflammation contributes to long-COVID-19 manifestation [117]. Another hypothesis that has been proposed is that the continuous shedding of the SARS-CoV2 virus is due to the diminished B and T cell count and function as the condition lingers [119]. This inflammatory dysfunction has been observed to take place about 2–6 weeks post infection, which certainly suggests adaptive immunity dysregulation [120]. Such inflammatory symptoms may present along with increased levels of pro-inflammatory markers (e.g., interleukin-6 (IL-6), C reactive protein (CRP), ferritin, and D-dimer), with no respiratory problems but with neurological and cardiovascular as well as gastrointestinal symptoms [119,120]. To confirm this persistent inflammation in long COVID-19, some researchers have performed radiological assessments of the implicated tissues and bones for Fluorine 18 fluorodeoxyglucose (FDG). FDG uptake identifies the foci of infection and correlates with the metabolic rate of the cells [121]. FDG positron emission tomography (PET) can indicate disease severity and spread and help correlate findings with treatment response [121]. Patients with long COVID-19 have shown increased and chronic inflammation in bones and vessels at least 4 weeks post-infection [119,121]. That said, it is important to note that although different studies have found increased levels of pro-inflammatory markers in long-COVID-19 patients, others have not found the same correlation [119,121]. Such findings explain the current lack of a resolved understanding of long COVID-19, which varies in terms of timeline, manifestation, symptoms, and, therefore, treatment [122]. This variation in clinical presentations has not been found to be significantly correlated with age, acute infection severity, or gender and race, which presents health practitioners with problems when predicting the condition or delivering a prognosis [123]. 

Nevertheless, attempts have been made to find out about associations with long COVID-19 that can predict a chronic pathology. The Institute for Systems Biology conducted a longitudinal mutli-omic systems biology investigation of 200 subjects 2–3 months post-acute COVID-19 infection [124]. The findings of this study reveal some factors significantly associated with long COVID-19, which include the following: pre-existing diabetes mellitus, high-level SARS-CoV-2 viremia, autoantibodies (including those targeting the interferons), and Epstein–Barr virus (EBV) reactivation during acute infection [124]. This study found that if these factors are present, then it is highly likely that COVID-19 patients will develop long COVID-19. Among the most devastating long-COVID-19 symptoms are the cardiovascular problems that develop following acute infection. One report cited that immunothrombosis and venous thromboemboli are critically dangerous in long-COVID-19 patients. This certainly makes examining predictive parameters important to preventing possible deaths due to such symptoms. The immune system and the hemostatic system are linked, allowing multiple factors to cause immunothromobosis in long COVID-19 [3,107,110,111]. In the occurrence of this immunothrombosis, the following factors have been implicated: endothelial inflammation, microthrombi formation, and the disruption of the intercellular junction [125]. Increasing levels of cytokines during the infection, along with activated platelets, are found to be associated with coagulopathy in long COVID-19, as it creates the proinflammatory environment of immunothrombosis, which is primarily a host defense mechanism that becomes altered, forming immunologically mediated thrombi that influence the microvasculature [125]. Dysfunctional endothelial cells (ECs) disturb the control of coagulation and anti-coagulation systems, which ultimately results in the increased coagulation seen in COVID-19 and long-COVID-19 conditions [126]. In addition to this, tissue factor (TF) is found to be released by ECs under inflammatory conditions, which, once in the blood stream, activates the coagulation system [127,128]. The same has been found in monocytes during inflammation and both result in increased mortality [129]. The different factors implicated in the immune dysregulation causing long COVID-19 call for more studies to investigate the stages at which each factor is important and when it combines with other parameters to manifest the pathology. Immune pathophysiology is essential to not only understanding the condition, but also to managing it efficiently.

**Table 1:** Proposed definitions and categories of long COVID-19 (2).

Description	Terms	References
Symptoms lasting 4 weeks post-acute infection	Long COVID	[104]
Symptoms 3 months post-acute infections	Post COVID	[130]
Long-term COVID-19 is said to be cyclical, progressive, and multiphasic	Long COVID	[131]
Multi-organ indications that continue for months after acute COVID-19	Long-hauler COVID-19	[132,133]
Chronic COVID syndrome	Long-COVID	
Symptoms lasting more than 100 days	Long-haul COVID	[134]
	Long-tail COVID	
Symptoms lasting more than 2 months	Long COVID	[135,136]
Symptoms to last more than 4 weeks	Late sequelae of SARS-CoV-2 infection	[137,138]
	Long-haulers	
	Long-COVID	
Symptoms persist more than 4 weeks after acute infection diagnosis	Post-acute COVID-19 syndrome	[130]
Symptoms continue for 5–12 weeks	Acute post-COVID symptoms	[139]
Symptoms continue for 12–24 weeks	Long post-COVID symptoms	
Symptoms continue for >24 weeks	Persistent post-COVID symptoms	
Symptoms continue for 1–3 months	Post-acute COVID-19	[106,140]
	On-going symptomatic COVID-19	
Symptoms continue more than 3 months	Chronic COVID-19	
	Long COVID	
	Post-COVID-19 syndrome	

## 5. Potential Preventive and/or Therapeutic Effects of Prebiotic- and Probiotic-Related Strategies in COVID-19

Probiotics are live microbes that confer beneficial effects on the host, when administered in appropriate quantities [141]. Prebiotics are defined as “substrates that are selectively utilized by host microorganisms conferring a health benefit [142]”. Interventions targeting the gut microbiome may have systemic beneficial effects in patients with COVID-19 [143]. Evidence indicates that viral infections in the respiratory tract initiate a disturbance in the gut microbiota; in patients with COVID-19, the gut microbiota is transformed via severe hypoxemia [144,145].

**Figure 5:**
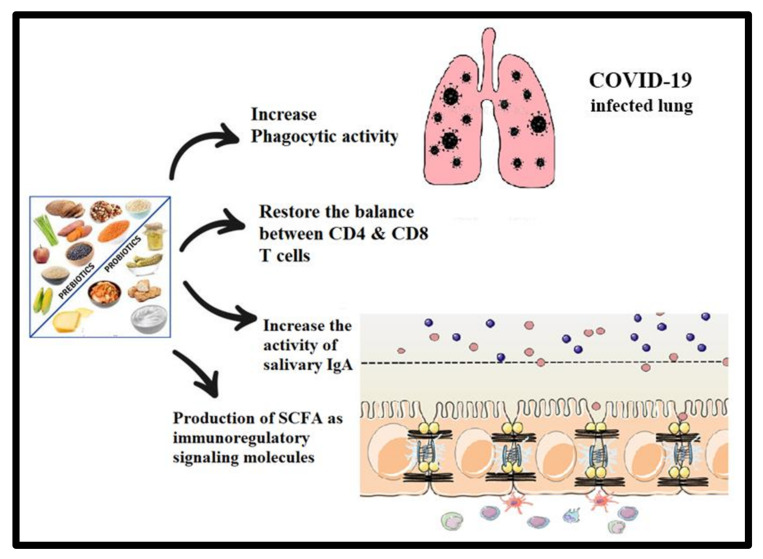
Interactions between the human gut and lung and potential positive immune responses triggered by probiotics and prebiotics.

Probiotics and prebiotics are the two components in our diet that can affect the microbiome. Nutritional status and diet play a crucial role in COVID-19, predominantly owing to the bidirectional interaction between the lungs and the gut microbiota [146]. Figure 5 depicts the interactions between the human gut and lungs and the potential positive immune responses triggered by probiotics and prebiotics. Both can enhance the phagocytic activity of macrophage cells [147], balance T cell immunity in favor of a more regulatory status [148], increase the activity of salivary IgA [149], and exert immunoregulatory extracellular and intracellular functions through the production of SCFAs as important signaling molecules [150]. The manipulation of the gut microbiota using prebiotics and probiotics represents a promising therapeutic approach to lung diseases in clinical research [147] (Figure 5).

The China National Health Commission and National Administration of Traditional Chinese Medicine Guidelines have recommended the consumption of probiotics along with conventional therapies in patients with severe COVID-19 infection, in order to improve the balance of intestinal flora and prevent secondary bacterial infections [148].

Probiotics may alter the composition of the gut microbiota and play a crucial role in maintaining the ecosystem of the gut microbiota [149]. Although the immune responses caused by bacteria are relatively different from those caused by the virus, numerous clinical studies have concluded that probiotics contribute to the fight against COVID-19 [150]. Apart from the improvements in the intestinal microbial balance, recent evidence indicates that probiotics can also confer beneficial effects on the host through modulating host immune functions [151]. Some studies have reported on the potential of probiotics to interact with angiotensin converting enzyme II, a host entry receptor of severe acute respiratory syndrome coronavirus 2 (SARS-COV 2) [143]. For example, several probiotics (mainly probiotic lactic acid bacteria) have been reported to release peptides with high affinity for the angiotensin-converting enzyme during milk fermentation [152]. Similarly, probiotics may also improve respiratory tract infections through the angiotensin converting enzyme II pathway [153]. Probiotics also improve the levels of natural killer (NK) cells, type I interferons, T and B lymphocytes, and antigen-presenting cells (APC) in the lung immune system [144]. NK cells play an important role in the early immune response against viral infections, predominantly via the clearance of viral infections. A previous study demonstrated that probiotics alter the expression of interleukin-10 and reduce the expression of inflammatory cytokines [154].

A recently published study in China revealed that probiotics have the ability to moderate immunity and reduce the incidence of secondary infection in patients with COVID-19 [153]. Baud and colleagues in 2020 [144] reported the following probiotics as potentially able to reduce the burden of COVID-19: Bifidobacterium bifidum, Lactobacillus plantarum, *Pediococcus pentosaceus*, *Leuconostoc mesenteroides*, *Bifidobacterium longum*, *Lactobacillus rhamnosus*, *Lactobacillus gasseri*, *Bifidobacterium breve*, and *Lactobacillus casei* [144]. A previous study in China concluded that COVID-19 infection affects the intestinal microbiota balance, based on the observation of reduced counts of Bifidobacterium and Lactobacillus strains in COVID-19 patients [155]. Probiotic strains restore the gut microbiota, decrease the translocation of pathogenic bacteria across the gut mucosa, maintain a healthy gut–lung axis, and lessen the incidence of secondary bacterial infection [156]. *Enterococcus* sp., *Bacillus* sp., *Bifdibacterium* sp., *actobacillus* sp., *Streptocococus* sp., and *Pediococcus* sp. are the most frequently used species in the preparation of probiotics [157,158].

Prebiotics include polyunsaturated fatty acids, resistant starch, arabinooligosaccharides, oligosaccharides, fructans, oligosaccharides, galactomannan, psyllium, lactosucrose, lactobionic acid, and polyphenols [142,159]. Foods that contain prebiotics, such as fiber, oligosaccharides, and polyphenols, can improve the growth of bacteria [160,161]. Prebiotics modulate the gut microbiota in a similar manner to probiotics, thus, inhibiting pathogens and stimulating the immune system. Likewise, prebiotics, via direct and indirect mechanisms, confer beneficial alterations upon the immune system and the host’s health [162]. In addition, they selectively stimulate the favorable growth and enhance the activities of probiotic bacteria [163]. Prebiotics have potential effects against COVID-19 infection by enhancing the growth and survivability of probiotics. Moreover, prebiotics provide energy for the growth of probiotics [143]. Prebiotics may also have a potential effect on gastrointestinal symptoms caused by COVID-19 by blocking angiotensin-converting enzymes [145].

Prebiotics apparently reduce the levels of the proinflammatory interleukin 6, which seems to be the leading cause of the hitherto described grave prognosis of COVID-19 and improve the levels of anti-inflammatory interleukin 10 [164]. The concurrent use of prebiotics and probiotics is crucial for the treatment of COVID-19 infection [165]. However, there is limited evidence regarding the effectiveness of prebiotics in COVID-19 infection, unlike the case for probiotics.

## 6. Conclusions

According to the previous findings, it can be concluded that a patient’s chance of acquiring “long COVID-19” after infection with SARS-CoV-2 may be influenced by the composition of their gut flora. Thus, an altered gut microbiota or dysbiosis can act as modulators of systemic inflammatory activity and can affect different organs through the multiple gut–organ axis. Increased gut permeability, or leaky gut, allows the entrance of bacterial metabolites and toxins into the circulatory system and further worsens the systemic inflammatory response, leading to different COVID-19 complications. Thus, the adjustment of the gut microbiome with probiotics could be an alternative strategy for boosting immunity, treating COVID-19, and protecting against the development of post-acute COVID-19 syndrome.

## 7. Future Direction

Based on the reviewed literature, a healthy gut microbiota composition during hospitalization is associated with a more favorable clinical presentation of COVID-19. Further studies are required to explore the direct connection between gut bacterial profiles and long-COVID-19 complications and to consider microbial configurations for prognostic and therapeutic strategies in clinical practice.
